# Peripheral T cell lymphopenia in COVID-19: potential mechanisms and impact

**DOI:** 10.1093/immadv/ltab015

**Published:** 2021-07-02

**Authors:** Sifan Zhang, Becca Asquith, Richard Szydlo, John S Tregoning, Katrina M Pollock

**Affiliations:** Department of Infectious Disease, Imperial College London, London, UK; Department of Infectious Disease, Imperial College London, London, UK; Centre for Haematology, Department of Immunology and Inflammation, Imperial College London, London, UK; Department of Infectious Disease, Imperial College London, London, UK; Department of Infectious Disease, Imperial College London, London, UK

**Keywords:** CD4 cell, CD8 cell, SARS-CoV-2 virus, COVID-19, T cell biology

## Abstract

Immunopathogenesis involving T lymphocytes, which play a key role in defence against viral infection, could contribute to the spectrum of COVID-19 disease and provide an avenue for treatment. To address this question, a review of clinical observational studies and autopsy data in English and Chinese languages was conducted with a search of registered clinical trials. Peripheral lymphopenia affecting CD4 and CD8 T cells was a striking feature of severe COVID-19 compared with non-severe disease. Autopsy data demonstrated infiltration of T cells into organs, particularly the lung. Seventy-four clinical trials are on-going that could target T cell-related pathogenesis, particularly IL-6 pathways. SARS-CoV-2 infection interrupts T cell circulation in patients with severe COVID-19. This could be due to redistribution of T cells into infected organs, activation induced exhaustion, apoptosis, or pyroptosis. Measuring T cell dynamics during COVID-19 will inform clinical risk-stratification of hospitalised patients and could identify those who would benefit most from treatments that target T cells.

## Graphical Abstract

**Figure:** In this review, we demonstrate that CD4^+^ and CD8^+^ T cell lymphopenia is a feature of severe COVID-19 compared with moderate disease in hospitalised patients. Pathology data indicate T cell infiltration into infected organs, particularly the lung. Potential mechanisms affecting T cells in severe disease due to SARS-CoV-2 infection include T cell redistribution and sequestration, pyroptosis, and apoptosis.

## Introduction

SARS-CoV-2 is the causative agent of the COVID-19 pandemic, responsible for a global health crisis unprecedented in recent times. A number of immunological, pathological, and histological studies indicate a role for T cells in the pathogenesis underlying COVID-19 [1–3]. However, the speed and spread of SARS-CoV-2, coupled with challenges in collecting experimental clinical evidence means that characterisation of immunopathogenesis has thus far been limited.

CD4^+^ and CD8^+^ T cells play key roles in containing and resolving viral infections. Suppression of T cell responses is associated with a failure to achieve sterilising immunity against viral infection, classically demonstrated by HIV infection [4, 5]. Viral pathogenicity can suppress T cell function through a number of mechanisms including direct and indirect cytotoxicity, organ-based sequestration, and suppression of both antigen recognition and the downstream effector mechanisms that contain infection [6–10]. Damage to the T cell compartment can also have longer term clinical and immunological sequelae, limiting responses to other pathogens even in recovered individuals [11–14].

Evidence from observational studies indicates that immunopathology is an important driver of the clinical features of severe COVID-19 [15, 16]. The mechanisms of this immunopathology in COVID-19 are unclear. An excess inflammatory response has been widely observed and is associated with the later stages of disease and multi-organ failure. Dysregulation of both CD8^+^ and CD4^+^ T cell circulation and function within specific tissues could contribute to this immune injury.

The quality and quantity of T cell function is important in all stages of SARS-CoV-2 infection, once viral replication is established, including viral containment, the resolution of infection and recovery. Severe COVID-19 therefore mainly represents a failure of normal T cell function to contain and resolve SARS-CoV-2 infection. We systematically reviewed clinical observational data from histopathology and immunological studies of autopsies and *in vivo* clinical observational cohorts to identify whether the data gathered so far supports this idea.

## Search strategy and selection criteria

**Figure 1.**
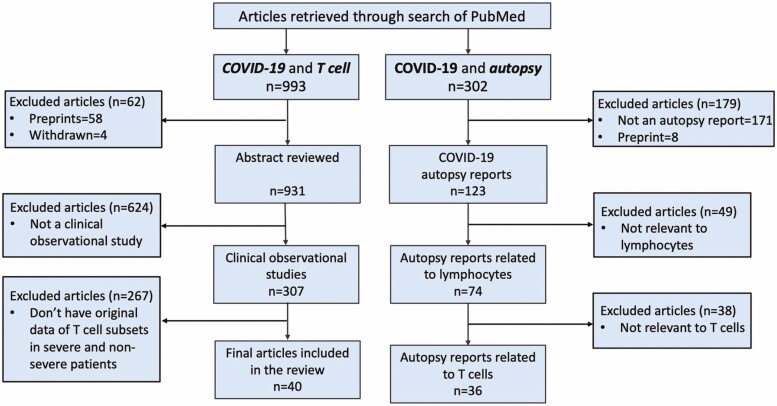
Flow chart of the study selection process.

Observational studies for this review were identified through a search of PubMed for articles published from 1 December 2019 to 31 December 2020, by use of the terms ‘COVID-19’ and ‘T cells’. Articles published in English and Chinese were included. Observational studies were selected if they reported original T cell counts in patients with severe and non-severe COVID-19. The selection process is shown as a flow chart (Fig. 1).

Autopsy studies for this review were identified through a search of PubMed for articles published from 1 December 2019 to 31 December 2020, by use of the terms ‘COVID-19’ and ‘Autopsy’. Articles published in English and Chinese were included. The inclusion criteria for post-mortem studies were measurement of T lymphocytes in patients who died of COVID-19 by haematoxylin and eosin (H&E) or immunohistochemistry (IHC) staining. The selection process is shown as a flow chart (Fig. 1).

Clinical trials for this review were identified through a search of ClinicalTrials.gov database for trials started from 1 December 2019 to 31 December 2020, by use of the terms ‘COVID-19’ and ‘CD147/IL-6/CCR5/PD-1/mTOR’. Phase 1 clinical trials were excluded; phase 2, 3, and 4 clinical trials were included.

## Clinical classification of COVID-19 and data extraction

Clinical descriptions in the eligible publications, which were from China, the Republic of Korea, Italy, France, Poland, Turkey, and Spain, followed broadly similar guidelines to classify patients with COVID-19 by disease severity. Four categories of COVID-19 disease were described, all of which had confirmed COVID-19 by polymerase chain reaction (PCR) testing or suspected COVID-19 based on clinical diagnostic criteria such as the Chinese Clinical Guidance of COVID-19 Pneumonia Diagnosis and Treatment [17] (Supplementary Table 1). Cases were asymptomatic, mild-moderate, severe, or critical. Mild-moderate cases had fever and respiratory symptoms such as dry cough, nasal obstruction, sore throat; severe cases were defined as those with pneumonia causing respiratory compromise and an oxygen saturation ≤93% when breathing room air at rest, and critical illness was defined as cases of COVID-19 where invasive ventilation was required. A summary of clinical features measured in the included publications is shown in the Supplementary Table 1. The reported CD3^+^ cell count (×10^6^ cells/L), CD4^+^ cell count (×10^6^ cells/L), CD8^+^ cell count (×10^6^ cells/L), and CD4:CD8 ratio were extracted from the selected publications. Cases that were admitted to intensive care unit (ICU), did not survive or were designated as critical or severe in the original publication were allocated to the severe group, cases that did not meet these criteria were allocated as non-severe. Infection with SARS-CoV-2 was confirmed in the autopsy cases by PCR, spike protein under transmission electron microscopy, IHC, or RNA hybrid assay.

## Peripheral T cell lymphopenia in severe COVID-19

**Figure 2.**
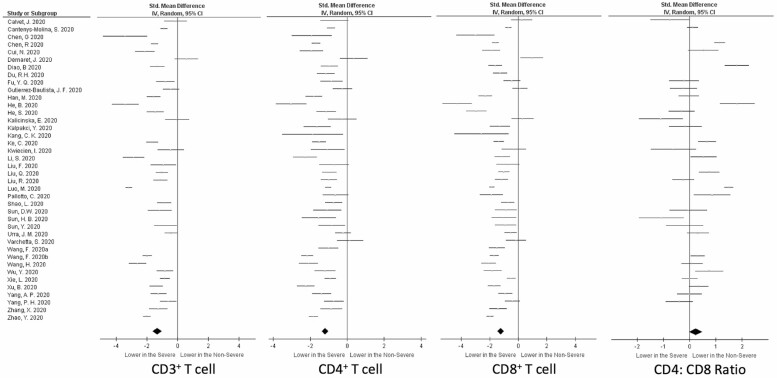
Forest plot of T cell subsets in patients with severe and non-severe COVID-19. CD3^+^, CD4^+^ and CD8^+^ T cell counts are significantly lower in patients with severe COVID-19 compared with those in patients with non-severe COVID-19 (*P* < 0.00001). The effect of severity of disease on the CD4:CD8 ratio was inconclusive (*P* = 0.08). Black diamond represents test for overall effect of 40 studies.

Data on peripheral blood white cell counts and immune cell subsets have been widely collected during hospital admission for COVID-19. Forty studies have overwhelmingly reported that peripheral T cell lymphopenia was worse in patients with severe COVID-19 compared with those with mild disease (Table 1). Although a wide range of T cell counts were reported with differences evident in standardised reported values for each study, the direction of change between severe and non-severe COVID-19 in these studies was consistent (Table 1). For all studies reporting CD3, CD4, and CD8 counts, the standardised mean difference in severe versus non-severe COVID-19 was significantly lower in severe disease (*P* < 0.00001 for all three; Fig. 2). For studies which only presented the median and interquartile range (27/40), mean and standard deviations (SD) were estimated using mathematical methods [18].

**Table 1.** Raw data of T cell subsets in patients with severe and non-severe COVID-19

Paper	Location	N (Non-severe: severe)	CD3^+^ T cell count (×10^6^/L)		CD4^+^ T cell count (×10^6^/L)		CD8^+^ T cell count (×10^6^/L)		CD4:CD8 Ratio		Reference number (DOI)
			Non severe	Severe	Non severe	Severe	Non severe	Severe	Non severe	Severe	
Calvet, J. 2020 [19]	Spain	17:13	725 (497–1119)	647 (375–1113)	545 (445–767)	278 (178–663)	253 (145–319)	237 (87–586)	3.12 (1.58–3.99)	1.72 (0.78–2.52)	10.3390/v12111277
Cantenys-Molina, S. 2020 [20]	Spain	590:112	662 (464–890)	363 (251–581)	412 (288–568)	242 (154–353)	213 (137–331)	116 (67–210)	1.90 (1.33–2.71)	1.98 (1.18–3.14)	10.1111/cei.13547
Chen, G. 2020 [21]	China	10:11	640.5 (588.3–789.5)	294.0 (169.3–415.3)	381.5 (255–451)	177.5 (104–249.8)	254 (183.3–312.8)	89 (61.5–130.3)	N/A	N/A	10.1172/JCI137244
Chen, R. 2020 [22]	China	345:155	662.00 (452.00–950.00)	367.5 (210.00–556.00)	386 (287.50–691.00)	226.5 (144.25–362.25)	252.5 (168.75–371.75)	126.5 (61.25–164.50)	1.52 (1.17–2.36)	1.94 (1.55–2.58)	10.1016/j.jaci.2020.05.003
Cui, N. 2020 [23]	China	118:13	1047 (760–1330)	122 (57–322)	610 (446–808)	93 (30–225)	336 (245–449)	51 (17–122)	1.88 (1.36–2.52)	2.01 (1.32–4.04)	10.3389/fcimb.2020.595333
Demaret, J. 2020 [24]	France	10:24	1000 (700–1400)	1300 (1000–1800)	700 (400–900)	700 (600–1100)	300 (200–500)	500 (400–600)	N/A	N/A	10.1002/cti2.1217
Diao, B. 2020 [25]	China	479:20	652 (351–977)	261 (157–457)	342 (192–559)	198 (100–279)	208 (118–356)	64.3 (40.7–160)	1.60 (1.17–2.28)	2.43 (1.50–4.25)	10.3389/fimmu.2020.00827
Du, R. H. 2020 [26]	China	158:21	N/A	N/A	128.3 (73.5–201.7)	68 (55.1–148.8)	104.5 (58.5–142.7)	47.9 (25.4–73.8)	NA	NA	10.1183/13993003.00524-2020
Fu, Y. Q. 2020 [27]	China	71:14	609.00 (410.00–905.00)	339.50 (217.50–524.25)	368.00 (246.00–549.00)	203.00 (126.50–284.25)	205.00 (111.00–303.00)	145.00 (70.00–213.00)	1.93 (1.26–2.68)	1.59 (1.13–2.47)	10.1371/journal.pone.0240751
Gutierrez-Bautista, J. F. 2020 [28]	Spain	100:17	859 (549–1278)	630 (500–876)	387 (258–573)	313 (262–467)	172 (101–303)	167 (123–334)	2.41 (1.50–3.53)	1.96 (1.04–3.03)	10.3389/fimmu.2020.596553
Han, M. 2020 [29]	China	122:32	864 (598–1125)	477 (337.675)	462 (314–621)	221 (185–407)	326 (224–478)	172 (119–269)	1.37 (1.09–1.83)	1.36 (0.95–2.09)	10.1007/s00430-020-00693-z
He, B. 2020 [30]	China	32:21	794 (586–1112)	221 (168–414)	433 (318–651)	146 (107–277)	297 (230–388)	59 (33–109)	1.45 (1.24–1.80)	2.38 (1.62–4.63)	10.3389/fimmu.2020.02075
He, S. 2020 [31]	China	48:25	821.86 (526.19)	465.28 (505.67)	475.68 (263.57)	277.07 (306.09)	302.35 (256.25)	185.75 (166.3)	1.68 (0.9)	1.46 (0.96)	10.1016/j.ijid.2020.06.059
Kalicinska, E. 2020 [32]	Poland	11:16	530 (360–980)	540 (175–1080)	320 (200–530)	185 (95–525)	200 (98–380)	320 (60–630)	1.65 (1.18–2.75)	1.01 (0.59–1.2)	10.1016/j.tranon.2020.100943
Kalpakci, Y. 2020 [33]	Turkey	20:20	N/A	N/A	958.83 ± 416.24	395.45 ± 237.59	504.15 (313.61–786.22)	192.00 (135.52–261.80)	1.57 (1.38–2.85)	1.81 (1.16–3.13)	10.1590/1806-9282.66.12.1666
Kang, C. K. 2020 [34]	Republic of Korea	8:3	N/A	N/A	435.5 ±57.5	306.2 ±79.0	296.1 ±60.9	143.2 ±13.1	N/A	N/A	10.1016/j.ijid.2020.05.106
Ke, C. 2020 [35]	China	148:46	965.06 ±421.16	322.88 ±223.97	612.47 ±277.33	218.31 ±179.59	316.26 ±181.77	90.31 ±76.92	2.28 ±1.39	3.33 ±2.11	10.1016/j.medcli.2020.06.055
Kwiecien, I. 2020 [36]	Poland	9:14	951 (683–2253)	691 (524–1416)	619 (533–1210)	319 (239–584)	313 (225–862)	330 (160–549)	2.1 (1.4–3.6)	1.3 (0.6–2.4	10.3390/cells9122615
Li, S. 2020 [37]	China	43:26	991 (740–1154)	378 (258–576)	544 (364–667)	199 (128–325)	417 (309–539)	134 (91–237)	1.18 (0.96–1.58)	1.40 (0.79–2.08)	10.1172/jci.insight.138070
Liu, F. 2020 [38]	China	32:8	857.74 ± 283.59	582.25 ± 305.80	454.16 ± 193.24	317.63 ± 162.30	385.74 ± 142.82	273.63 ± 168.54	N/A	N/A	10.1038/s41598-020-70387-2
Liu, Q. 2020 [39]	China	310:30	773 ± 549 678 (317.4–1105.1)	228 ± 168 170 (94.0–339.1)	457 ± 342 370 (182.8–651.0)	139 ± 98 115 (62.8–195.1)	297 ± 220 249 (118.8–425.1)	80.9 ± 97.7 56.8 (26.8–80.8)	1.71 ± 0.894 1.59 (1.06–2.04)	2.41 ± 1.28 2.25 (1.70–2.93)	10.1371/journal.pone.0239695
Liu, R. 2020 [40]	China	49:61	509 (336.5–873.5)	517 (376–651.5)	290 (172.5–506)	292 (237.5–399.5)	161 (101–302.5)	200 (93–261)	1.89 (1.25–2.64)	1.75 (1.09–2.75)	10.1016/j.cca.2020.05.019
Luo, M. 2020 [41]	China	817:201	611.01 (420.12–858.10)	391.20 (262.95–505.46)	367.99 (242.39–543.00)	245.00 (161.64–317.64)	203.98 (142.54–313.05)	96.89 (60.65–140.08)	1.70 (1.30–2.21)	2.37 (1.77–3.36)	10.1172/jci.insight.139024
Pallotto, C. 2020 [42]	Italy	25:13	N/A	N/A	461 (275–654)	348 (206–616)	184 (132–334)	100 (83–198)	2.4 (1.5–2.7)	2.8 (2.3–4)	10.1080/23744235.2020.1778178
Shao, L. 2020 [43]	China	104:25	824.54 ± 469.11	447.25 ± 177.74	504.39 ± 367.90	255.00 ± 88.37	327.29 ± 212.36	179.33 ± 119.96	N/A	N/A	10.3389/fmed.2020.00246
Sun, D. W. 2020 [44]	China	25:11	1089.680 ± 290.154	698.455 ± 393.675	686.960 ± 225.383	427.091 ± 251.712	359.84 ± 111.279	247.818 ± 153.683	1.993 ± 0.606	1.957 ± 0.905	10.1016/j.cca.2020.05.027
Sun, H. B. 2020 [45]	China	13:12	N/A	N/A	576 ± 234	293 ± 76	492 ± 214	323 ± 56	1.23 ± 0.37	0.91 ± 0.15	10.1371/journal.pone.0239532
Sun, Y. 2020 [46]	China	36:10	808.97 ± 371.22	522.57 ± 318.73	436.8 ± 225.08	257.8 6± 129.48	355.33 ± 166.86	205.14 ± 153.09	1.62 ± 1.86	1.28 ± 0.76	10.1016/j.jaut.2020.102473
Urra, J. M. 2020 [47]	Spain	145:27	701.0 ± 408.5	528.3 ± 350.9	395.9 ± 241.0	340.3 ± 251.9	287.6 ± 223.8	172.4 ± 123.9	1.9 ± 1.6	2.4 ± 1.4	10.1016/j.clim.2020.108486
Varchetta, S. 2020 [48]	Italy	17:15	N/A	N/A	280 (144–562)	291 (132–734)	189 (32–505)	125 (35–411)	N/A	N/A	10.1038/s41423-020-00557-9
Wang, F. 2020a [49]	China	33:32	N/A	N/A	363.7± 225.5	179.5± 110.1	206.3± 137.0	53± 35.14	N/A	N/A	10.1038/s41423-020-0483-y
Wang, F. 2020b [50]	China	253:70	1071 (772.5–1399)	529 (387.0–712.5)	596.5 (452.5–757.0)	302.0 (204.5–383.0)	402.5 (273.0–546.5)	201.0 (134.5–294.0)	1.48 (1.12–1.96)	1.61 (1.02–1.94)	10.1186/s12967-020-02423-8
Wang, H. 2020 [51]	China	48:47	774 (572–1095)	324 (195–455)	513 (304–625)	180 (109–274)	312 (197–423)	123 (71–179)	1.50 (1.11–2.04)	1.54 (1.04–2.51)	10.1016/j.intimp.2020.106683
Wu, Y. 2020 [52]	China	31:29	399 (324–626)	306 (167–422)	234 (156–401)	153 (102–289)	191 (125–288)	88 (45–147)	1.46 (0.78–2.11)	1.99 (1.28–3.75)	10.1128/mSphere.00362-20
Xie, L. 2020 [53]	China	322:51	1342.67 ± 493.06	938.79 ± 429.62	810.72 ± 321.02	530.61 ± 269.47	455.71 ± 198.07	357.58 ± 198.21	2.02 ± 0.96	1.87 ± 1.27	10.1177/1753466620942129
Xu, B. 2020 [54]	China	117:28	894.50 (662.75–1192.00)	593.00 (412.00–725.00)	573.50 (426.75–771.00)	299.00 (249.00–460.00)	323.50 (232.75–448.75)	188.00 (134.00–274.00)	1.68 (0.96–2.18)	1.96 (1.02–2.70)	10.1016/j.jinf.2020.04.012
Yang, A. P. 2020 [55]	China	69:24	763.8 ± 483.3	222.2 ± 195.2	448.7 ± 254.9	132.6 ± 98.5	264.6 ± 217.4	83.9 ± 97.2	2.01 ± 0.98	2.00 ± 0.97	10.18632/aging.103255
Yang, P. H. 2020 [56]	China	22:48	975.00 (685.00–1365.00)	731.00 (377.25–1019.50)	540.00 (364.00–724.00)	377.00 (196.00–513.00)	417.00 (295.00–545.00)	326.50 (137.00–490.50)	1.41 (1.07–1.59)	1.13 (0.85–1.53)	10.1186/s40249-020-00780-6
Zhang, X. 2020 [57]	China	293:12	778 (553–1041)	500 (379–705)	455 (314–650)	332 (226–541)	265 (171–393)	133 (102–199)	N/A	N/A	10.1038/s41586-020-2355-0
Zhao, Y. 2020 [58]	China	414:125	814 (516–1088)	277 (163.5–430)	473 (291–657.75)	172 (99.5–267.5)	262.5 (163–405.25)	84 (39.5–155.5)	N/A	N/A	10.1186/s40249-020-00723-1

Data are presented as median (IQR) and/or mean ± SD.

Where reported (in 30/40 studies), data on the CD4:CD8 ratio were more variable compared with findings for the lymphocyte subsets (Table 1). Overall, the CD4:CD8 ratio was not significantly different in those patients with non-severe disease compared with patients with severe disease (*P* = 0.08), and the direction of change varied across the studies (Table 1, Fig. 2). Normal CD4:CD8 ratio is variously described as ≥ 1 or ≥ 1.2 and decreases with age [59]. Only one study reported a CD4:CD8 ratio <1.2, in either severe or non-severe COVID-19, the CD4:CD8 ratio therefore remained within normal range in both non-severe and severe COVID-19. Taken together, and compared with non-severe COVID-19, there is mounting evidence that severe COVID-19 disease is associated with a reduced frequency of CD3^+^ T cells affecting both CD4^+^ and CD8^+^ T cells in the peripheral circulation.

Given that lymphopaenia is known to be a feature of viral infection, rather than unique to severe COVID-19 we searched for observational studies that had measured CD4 and CD8 counts in other respiratory viral infections. Only two studies that reported comparative lymphocyte levels comparing between severe and non-severe infections were found, one in influenza [60] and one for SARS-CoV-1 [61]. Both indicated acute lymphopenia in severe disease (Supplementary Table 2). One study has compared lymphocyte levels between patients with SARS-CoV-2 and influenza, and the counts were similar [62]. This suggests that lymphopenia may be a common feature of severe respiratory viral infections and not unique to SARS-CoV-2 and therefore strategies that target it may be more broadly beneficial.

## Clinical, virological, and immunological significance of T cell lymphopenia in COVID-19

Lymphopenia could be a risk factor for severity, mortality, and poor prognosis in COVID-19, with T cells the most affected compared with B cells and natural killer (NK) cells [63, 64]. T cell counts negatively correlated with survival and CD4^+^ and CD8^+^ T lymphocytes decreased significantly in patients with severe disease [40]. In the early stage of the COVID-19 outbreak, a hospital in Wuhan studied lymphocyte subsets in 27 patients; lymphopenia was common (70.4%, 19/27) in severe cases, and T lymphocytes decreased more than B lymphocytes. Interestingly, CD4^+^ and CD8^+^ T cells but not B lymphocytes or NK cells, were significantly elevated after treatment of symptomatic disease suggesting that clinical and immunological recovery may be correlated and can be measured by an improving peripheral CD4 and CD8 count [65]. In another study, although CD4:CD8 ratio remained in the normal range, CD8^+^ T lymphocytes were the most improved subsets after treatment [66]. While only one study reported that CD4^+^ T cell count was independently associated with ICU admission [67], several results highlighted the role of CD8^+^ T cells in COVID-19. CD8^+^ T cell lymphopenia was analysed as an independent predictor for the prognosis of COVID-19 [26, 46]. Notably, an association of decreased lymphocytes with disease severity, has been observed suggesting that lymphopenia is an important predictive factor for the severity of COVID-19 [57,68,69].

The nature of the association between lymphopenia and failure of viral containment has not been established, however a negative correlation between T cell count and virus detection could indicate that T cells contribute to the limitation of viral load. Peripheral blood lymphocyte counts on admission were significantly and negatively correlated with SARS-CoV-2 nucleic acid-positive duration in 18 patients. Amongst lymphocytes, the T cell count but not B cell or NK cell count that negatively correlated with nucleic acid-positive duration [70]. Another study which analysed T cells in 66 recovered COVID-19 patients, found that CD4^+^ T cell count was predictive of the duration of viral RNA detection in the stool sample [71]. These data suggest that depletion of T lymphocytes is associated with a delay in viral clearance; however, the direction of causality is yet to be established.

While clinical data indicate T cell lymphopenia is associated with persistent SARS-CoV-2 viraemia, some studies also indicate that infection may alter the quality and phenotype of the CD4^+^ T cell response. Surprisingly, a higher than normal naive-to-memory CD4^+^ T cell ratio has been observed suggesting an impact on the differentiation of naive to memory T cells in COVID-19 patients [64]. There was a lower frequency of regulatory T cells (T regs) in patients with severe disease compared with those with mild disease [34]. Patients had reduced levels of CD4^+^ Th1 cells [72], with a reported Th2 skewed response in peripheral blood smears from ICU patients [73]. An upregulated Th17 response was found in 39 COVID-19 patients [74]. Bioinformatics analysis shows that genes involved in Th17 cell differentiation were enriched in patients with both mild and severe COVID-19 [75]. Altogether these observations of skewed T cell helper phenotypes suggest that the inhibition of Treg-induced anti-inflammatory responses and increased Th2 or Th17 responses, which are inflammatory without necessarily leading to viral control, could be involved in the pathogenesis of COVID-19 [76].

## Possible mechanisms for T cell lymphopenia

Dissection of the mechanism for the universal finding of peripheral T cell lymphopenia in SARS-CoV-2 infection particularly in severe COVID-19, will be important for the development of treatments and identifying who is at risk of severe disease.

### SARS-CoV-2 infection of T cells

It is not yet clear whether SARS-CoV-2 can cause cytopathology through directly infecting T cells. Viral gene and angiotensin-converting enzyme 2(ACE2) expression were not detectable in the peripheral blood mononuclear cells (PBMCs) of COVID-19 patients [77]. Findings that the spike protein can bind to CD147 also known as basigin, and mediate viral invasion have not been corroborated [78, 79]. More recently a study has suggested that SARS-CoV-2 could interact with the CD4 molecule and that CD4-positive cells are permissive to viral infection [80]. However *in vivo* infection of CD4^+^ but not CD8^+^ T cells is not supported by published studies. Given the very early and conflicting nature of the data, caution is needed when interpreting these reports.

### T cell redistribution

Although SARS-CoV-2 replication occurs principally in the lung, in severe cases, patients present with multi-organ failure in the late stage. This begs the question as to whether this is due to direct viral infection of these organs or due to an indirect effect of viral replication in the lung. To investigate this, we evaluated 123 autopsy case series of SARS-CoV-2 patients. In the respiratory tract, lung congestion, patchy lesions, diffuse alveolar damage, widespread vascular thrombosis, and new vessel growth were distinct characteristics of COVID-19 [81, 82]. Of significance for the interaction of the virus with T cells, 74 out of 123 studies reported mild-to-prominent infiltration of lymphocytes into organs by H&E staining, 36 out of 74 studies additionally confirmed T cell infiltration by IHC staining (Fig. 1). Both T cell infiltration and viral infection, as confirmed by presence of spike protein and/or viral nucleic acid, was observed in the heart [83], spleen [84], pharynx [82, 85], liver [83], and kidney [86]. Activated T cells in lymph nodes have also been reported [87]. In the spleen, the commonly reported lymphoid hypoplasia and lymphocytic depletion, especially CD8^+^ T cells indicates a decrease in the source of circulating lymphocytes [88]. The absence of germinal centres in hilar and posterior mediastinal lymph nodes suggests that the differentiation and maturation of B cells is also impacted [87].

**Figure 3.**
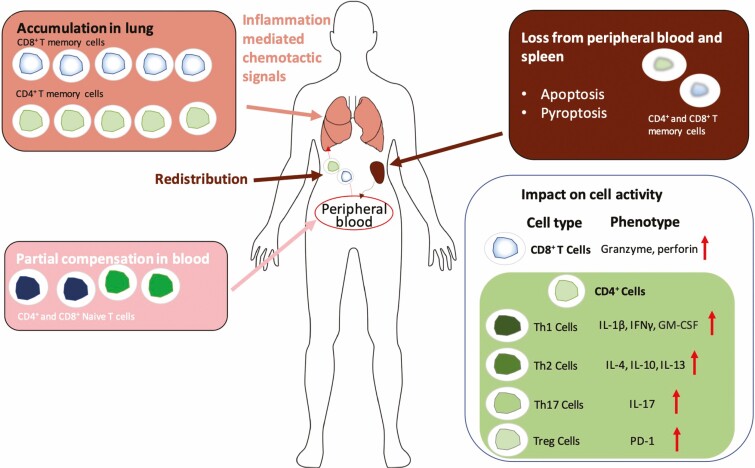
Hypothesis for peripheral T cell lymphopenia during SARS-CoV-2 infection and severe disease. According to experimental data from peripheral blood of patients with severe COVID-19, we propose two drivers for peripheral lymphopenia. Firstly, T lymphocytes in the periphery are attracted by chemokines released by infected cells and immune cells at the site of disease and migrate out of the periphery to infected organs, mainly the lungs. Secondly, functionally exhausted T lymphocytes and activation of Th1/Th2/Th17 responses at the site of disease, fail to achieve viral containment, and undergo cell death through a variety of mechanisms including apoptosis and pyroptosis. It is likely that interruption of the normal circulation of T cells is the key component in this cycle.

The movement of T cells into tissues could be driving some of the observed damage. Cardiovascular changes reported were cardiomyocyte hypertrophy, degeneration, necrosis, congestion, and oedema of interstitial tissue [89]. H&E staining and caspase 3 staining support a model of inflammatory infiltration consistent with a pattern of endothelial apoptosis [90]. CD8^+^ T cell activation could contribute to cardiac injury in patients with severe disease [91]. The myocarditis found in histopathological studies could result from hyper inflammation of pathological T cells and cytokine storm [92]. Finally, the intense inflammation observed in severe COVID-19 could be associated with aberrant T-cell endothelial cell interactions leading to altered tethering and adhesion of cells [93]. Taken together, these autopsy findings indicate that normal T cell circulation is interrupted during severe disease due to SARS-CoV-2 infection (Fig. 3).

One potential driver for T cell redistribution contributing to the observed lymphopenia is the cytokine environment. Type I interferons are associated with SARS-CoV-2 infection, and in mouse models, lymphopenia was dependent on IFN signalling [94]. This may be a greater problem in the prolonged viral infection associated with severe COVID-19.

### T cell activation, exhaustion, and apoptosis

Given that lymphopenia was associated with higher viral loads, dysregulation of T cell circulation could be associated with antigen-driven over-activation and exhaustion, which may in turn lead to their absence in peripheral circulation. Data to support this hypothesis are scarce. The abnormal cytokine profile in COVID-19 has been well described and also in association with expression of apoptosis pathway transcripts in PBMC but more data are needed to demonstrate whether these are linked [77, 95]. Several studies have indicated expression of exhaustion and activation markers in severe COVID-19. Kinetic studies found that CD4^+^ and CD8^+^ T cells expressed higher levels of the exhaustion markers PD-1, Tim-3, and NKG2A in the symptomatic stage, compared with the prodromal and recovery stages [25, 96]. Analysis of a peripheral blood sample taken from a deceased patient showed that the remaining CD4^+^ and CD8^+^ T cells were hyperactivated, indicated by the expression of both CD38 and HLA-DR. The increased frequency of CCR6^+^ Th17 CD4^+^ T cells and perforin and granulysin positive CD8^+^ T cells suggests that T cell activation accounted for severe immune injury in this patient [97]. Downregulation of the costimulatory molecule CD28 in patients with severe disease also suggests activation of T cells might contribute to aberrant signalling [98]. Different levels of activation markers were observed in different classes of T cells, with a very high level of activation in CD8^+^ T cells observed compared to CD4^+^ T cells [99]. An incompetent or aberrant CD8^+^ T-cell response could limit antigen-specific immunity, and there is some evidence this might be occurring in older people, although our review has not demonstrated lymphopaenia to be uniquely CD8^+^ T cell biased [100, 101]. It was notable that in clonal analysis of CD8^+^ T cells, they were highly activated but not exhausted in COVID-19 patients compared with healthy controls [102]. While data are accumulating indicating T cell hyperactivation in COVID-19, whether this is mechanistically linked to lymphopaenia remains to be seen.

### Cell death

Finally, T cell lymphopenia could also be a function of SARS-CoV-2 infection-induced cell death. The cytokine storm produced by T cells and other inflammatory cells can promote apoptosis, pyroptosis, and necrosis of T cells in turn [25, 103]. A study of SARS-CoV found higher plasma Fas-ligand level in patients, which is associated with a higher level of caspase-3, which plays a key role in cell apoptosis, in CD4^+^ and CD8^+^ lymphocytes [104]. Since there are similarities between SARS-CoV-1 and SARS-CoV-2, it was proposed that lymphocyte apoptosis is one of the causes of lymphopenia in COVID-19. Genes involved in apoptosis and P53 signalling pathways were enriched in PBMCs and cells of bronchoalveolar lavage fluid sample in three COVID-19 patients, indicating cell apoptosis could contribute to lymphopenia [77].

Another form of inflammation induced cell death is pyroptosis, which is caspase-1 triggered cell death via cleavage of gasdermin family members. Pyroptosis is triggered by inflammatory cytokines. While no studies have directly reported T cell pyroptosis during COVID-19 infection, one study proposed a mechanism for cytokine induced cell death [105]. This cell death was linked to IFNγ and TNF, both of which are elevated in patients with severe COVID-19, so it could potentially be a mechanism. Type I interferons have also been shown to prime cells for Fas-mediated apoptosis [106] which may drive the cell death seen.

## Treatment strategies and ongoing clinical trials targeting T cells

The link between lymphopenia and severe disease opens up a number of therapeutic strategies, to target the different mechanisms driving the lymphopenia. Treatment strategies that have been considered include blocking SARS-CoV-2 from infecting T cells [78], inhibition of cytokine secretion [107–109], mitigation of T cell exhaustion [110, 111], blockade of the chemokine receptor CCR5 [112, 113], and normalisation of Th1/Th2/Th17 differentiation [114, 115]. Despite limited understanding of the pathogenesis and processes involving T cells in SARS-CoV-2 infection, some of these have already entered clinical trials (Table 2). Anti-IL-6 treatment has been tested in a number of different settings, with mixed results. A recently published study indicates that in critically ill patients anti-IL-6 receptor drugs can improve outcomes [116] but in other settings it has been less effective.

**Table 2.** Clinical trials using therapeutics targeting T cells

Rationale	Target	Drug	ClinicalTrials.gov Identifier
Proposed viral entry (mechanism to be confirmed)	CD147	Meplazumab (Anti-CD147 antibody)	NCT04275245 Phase 1, 2 NCT04586153 Phase 2, 3
Target a downstream component of aberrant immune cell communication Reduce cytokine-storm, inflammation and exhaustion	IL-6	Tocilizumab (Anti-IL-6R antibody)	ChiCTR2000029765 NCT04320615 Phase 3 NCT04330638 Phase 3 NCT04345445 Phase 3 NCT04347031 Phase 2, 3 NCT04349410 Phase 2, 3 NCT04356937 Phase 3 NCT04359095 Phase 2, 3 NCT04361032 Phase 3 NCT04372186 Phase 3 NCT04377750 Phase 4 NCT04380519 Phase 2, 3 NCT04381936 Phase 2, 3 NCT04403685 Phase 3 NCT04409262 Phase 3 NCT04412772 Phase 3 NCT04423042 Phase 3 NCT04424056 Phase 3 NCT04577534 Phase 3 NCT04600141 Phase 3 NCT04678739 Phase 3 NCT04730323 Phase 4
		Siltuximab (Anti-IL-6 antibody)	NCT04322188 Not shown NCT04329650 Phase 2 NCT04330638 Phase 3 NCT04486521 Not shown
		Clazakizumab (Anti-IL-6 antibody)	NCT04381052 Phase 2 NCT04343989 Phase 2 NCT04363502 Phase 2 NCT04348500 Phase 2 NCT04494724 Phase 2 NCT04659772 Phase 2
		Sarilumab (Anti-IL-6 Receptor antibody)	NCT04315298 Phase 2, 3 NCT04324073 Phase 2, 3 NCT04322773 Phase 2 NCT04327388 Phase 3 NCT04341870 Phase 2, 3 NCT04357808 Phase 2 NCT04357860 Phase 2 NCT04359901 Phase 2 NCT04661527 Phase 2
		Fluoxetine (SSRI inhibitor)	NCT04377308 Phase 4
		Ruxolitinib (*JAK* inhibitor)	NCT04331665 Not shown NCT04334044 Phase 1, 2 NCT04338958 Phase 2 NCT04348071 Phase 2, 3 NCT04348695 Phase 2 NCT04355793 Not shown NCT04359290 Phase 2 NCT04361903 Not shown NCT04362137 Phase 3 NCT04374149 Phase 2 NCT04377620 Phase 3 NCT04403243 Phase 2 NCT04414098 Phase 2 NCT04424056 Phase 3 NCT04477993 Phase 2, 3 NCT04581954 Phase 1, 2
Reduce aberrant T cell migration	CCR5	Maraviroc (CCR5 antagonist)	NCT04441385 Phase 2 NCT04475991 Phase 2 NCT04710199 Phase 2
		Leronlimab (Anti-CCR5 antibody)	NCT04343651 Phase 2 NCT04347239 Phase 2 NCT04678830 Phase 2
Limit T cell exhaustion	PD-1	PD-1 blocking antibody	NCT04268537 Phase 2
		Nivolumab (Anti-PD1 antibody)	NCT04356508 Phase 2 NCT04413838 Phase 2
Limit T cell exhaustion	mTOR	Rapamycin/ Sirolimus (mTOR inhibitor)	NCT04341675 Phase 2 NCT04482712 Phase 1, 2 NCT04461340 Phase 2
		RTB101 (PI3K/ mTOR) inhibitor	NCT04584710 Phase 2 NCT04409327 Phase 2

Dexamethasone, which suppresses the immune response via the glucocorticoid receptor, is the only drug so far to have shown to have consistent efficacy against COVID-19 [117]. Randomised clinical trials have shown that intravenous dexamethasone treatment significantly prolonged ventilator-free days [118] and reduced mortality [119]. A meta-analysis demonstrated lower 28-day all-cause mortality in the corticosteroid treatment group [120]. Despite clinical improvements, the side effect of dexamethasone including dampening viral clearance, suppressing bone marrow, and interrupting metabolism need attention [121]. The impact of dexamethasone on T cell circulation and function in COVID-19 will be important to study. Why dexamethasone, which acts pleiotropically has been more effective than targeted cytokine blocking drugs is not clear, there may be a goldilocks effect, where both too little and too great a cytokine response is detrimental, so dexamethasone dampens but does not completely block inflammation while specific cytokine drugs remove the beneficial role of the cytokine as well as the excess inflammation.

## Vaccines that induce a T cell response

Priming the T cell response prior to infection with vaccination is one strategy to improve protection from disease. To date, the published human clinical vaccine studies including T cell data have been largely phase I/II trials and are not designed to evaluate the protective role of T cells. Several of the studies have reported T cell responses after vaccination [122]. The mRNA vaccine BNT162b1-induced receptor binding domain-specific CD4^+^ and CD8^+^ Th1 responses with IFN γ production [123]. A combination of rAd26 and rAd5 vector-based vaccines induced proliferation of antigen-specific CD4^+^ and CD8^+^ T cells [124]. Adenovirus type-5-vectored COVID-19 vaccine successfully induced IFN γ-producing T cells in phase 1 and phase 2 clinical trial [125, 126]. ChAdOx1 nCoV-19 vaccine also elicited IFN γ-producing T cells to a similar degree [127]. Induction of a robust SARS-CoV-2-specific T cell memory response may be different in natural infection compared with immunisation, and the frequency of SARS-CoV-2-specific T cells may be contingent upon both vaccine design and whether the initial response to the prime dose has been boosted [128]. Larger, follow-up studies will be required to identify the role of vaccine-induced T cells in protection from infection and disease.

## Long COVID-19

The sequalae of infection with SARS-CoV-2 can be chronic and leave symptoms even in recovered nucleic-negative individuals such as fatigue, chest heaviness, and breathlessness [129]. These symptoms have been grouped together as long COVID. Although data are at present limited, persistence of symptoms is common, particularly in patients who are hospitalised, with only 13% of 143 patients symptom-free after discharge in one Italian study [130]. The role of T cells in this condition is yet to be identified, but aberrant T cell circulation, tissue infiltration, and immune damage during acute infection may all be important in determining longer-term outcomes.

## Conclusion

Peripheral circulation T cell lymphopenia affecting both CD4^+^ and CD8^+^ T cells is a universal finding in case series of COVID-19 and is associated with severe disease. Our findings are in line with other publications [131–134]. Current evidence from autopsy and *in vivo* studies indicates the most likely mechanism is the interruption of normal lymphocyte circulation and organ-based, and/or endothelial sequestration of T cells. This may be due to the intense inflammation caused by SARS-CoV-2 replication. Coupled with aberrant T cell function, T cell exhaustion, and persistent failure of viral containment, these findings indicate a self-amplifying inflammatory cycle in severe COVID-19, which should be prevented or interrupted with rationally designed vaccines and therapeutics.

## Supplementary material

Supplementary data are available at *Immunotherapy Advances* online.

Supplementary Table 1. Summary of studies included

Supplementary Table 2. Lymphocyte count of patients with SARS-CoV, MERS-CoV and Influenza. Cell count was shown as mean ± SD and/or median (IQR).

ltab015_suppl_Supplementary_Material

## Data Availability

No new datasets were generated for this manuscript.

## Abbreviations

ACE2: Angiotensin-converting enzyme 2
CCR5: C-C chemokine receptor type 5
CD: Cluster of differentiation
COVID-19: Coronavirus disease 2019
H&E: Haematoxylin and eosin
HIV: Human immunodeficiency virus
HLA-DR: Human leukocyte antigen – DR isotype
ICU: Intensive care units
IFN: Interferon
IHC: Immunohistochemistry
IL: Interleukin
mTOR: mechanistic target of rapamycin
NK: Natural killer
P53: Tumour protein P53
PBMC: Peripheral blood mononuclear cell
PCR: Polymerase chain reaction
PD-1: Programmed cell death protein 1
RNA: Ribonucleic acid
SARS-CoV: Severe acute respiratory syndrome coronavirus
Th: T helper
Tim-3: T-cell immunoglobulin mucin 3
Treg: regulatory T cell
