# Supplementary material for: Peripheral T cell lymphopenia in COVID-19: potential mechanisms and impact
Source: Immunother Adv. 2021 Jul 2;1(1):ltab015. doi: 10.1093/immadv/ltab015 (PMC9364037; doi:10.1093/immadv/ltab015)
Supplement: ltab015_suppl_Supplementary_Material [file ltab015_suppl_supplementary_material.docx]

# **Supplementary material**

**Supplementary table 1. Summary of studies included**

| **Author, year** | **Country** | **Study type** | **Severe group, number** | **Non-severe group, number** | **Case definitions (virologically confirmed or clinically confirmed)** |
| --- | --- | --- | --- | --- | --- |
| Chen, G. 2020 | China | Retrospective study | Severe, 11 | Moderate, 10 | Virologically confirmed |
| Chen, R. 2020 | China | Retrospective study | Severe, 155 | Mild/Moderate, 345 | Virologically confirmed |
| Diao, B. 2020 | China | Retrospective study | ICU, 20 | Non-ICU, 479 | Virologically confirmed |
| Du, R. H. 2020 | China | Prospective study | Deceased, 21 | Survivors, 158 | Virologically confirmed: 136  Clinically confirmed: 43 |
| Han, M. 2020 | China | Retrospective study | Severe, 32 | Mild, 122 | Virologically confirmed |
| He, B. 2020 | China | Retrospective study | Severe, 21 | Mild, 32 | Virologically confirmed |
| He, S. 2020 | China | Retrospective study | Severe, 25 | Mild, 48 | Virologically confirmed |
| Kang, C. K. 2020 | Republic of Korea | Retrospective study | Severe, 3 | Mild, 8 | Virologically confirmed |
| Ke, C. 2020 | China | Retrospective, case-control study | Died, 46 | Survival, 148 | Virologically confirmed: 173  Clinically confirmed: 21 |
| Li, S. 2020 | China | Retrospective study | Severe, 26 | Non-severe, 43 | Virologically confirmed |
| Liu, F. 2020 | China | Retrospective study | Severe, 8 | General, 32 | Virologically confirmed |
| Liu, Q. 2020 | China | Retrospective study | Death, 30 | Discharged, 310 | Virologically confirmed |
| Liu, R. 2020 | China | Retrospective study | Severe, 61 | Moderate, 49 | Virologically confirmed |
| Luo, M. 2020 | China | 2-center retrospective study | Non-survivors, 201 | Survivors, 817 | Virologically confirmed |
| Pallotto, C. 2020 | Italy | Retrospective multicentre study | Critical, 13 | Non-critical, 25 | Virologically confirmed |
| Shao, L. 2020 | China | Retrospective study | Severe, 25 | Ordinary, 104 | Virologically confirmed |
| Sun, D. W. 2020 | China | Retrospective study | Died, 11 | Discharged, 25 | Virologically confirmed |
| Sun, H. B. 2020 | China | Retrospective study | Severe, 12 | General, 13 | Virologically confirmed |
| Sun, Y. 2020 | China | Retrospective study | Severe, 10 | Moderate, 36 | Virologically confirmed |
| Urra, J. M. 2020 | Spain | Retrospective case-control study | ICU, 27 | Non-ICU, 145 | Virologically confirmed |
| Wang, F. 2020a | China | Retrospective study | Decease, 32 | Survivors, 33 | Virologically confirmed |
| Wang, F. 2020b | China | Retrospective study | Progressive, 70 | Stable, 253 | Virologically confirmed |
| Wang, H. 2020 | China | Retrospective study | Critical, 47 | Non-critical, 48 | Virologically confirmed |
| Wu, Y. 2020 | China | Retrospective study | Severe, 29 | Mild, 31 | Virologically confirmed |
| Xie, L. 2020 | China | Retrospective study | Severe, 51 | Non-severe, 322 | Virologically confirmed |
| Xu, B. 2020 | China | Retrospective, single-center study | Died, 28 | Discharged, 117 | Virologically confirmed |
| Yang, A. P. 2020 | China | Retrospective study | Severe (including critical illness), 24 | Non-severe, 69 | Virologically confirmed |
| Zhang, X. 2020 | China | Retrospective study | Severe, 12 | Mild, 293 | Virologically confirmed |
| Zhao, Y. 2020 | China | Retrospective, single-center study | Non-survivors, 125 | Survivors, 414 | Virologically confirmed |
| Gutierrez-Bautista, J. F. 2020 | Spain | Prospective study | ICU hospitalized patients, 17 | Non-ICU hospitalized patients, 100 | Virologically confirmed |
| Demaret, J. 2020 | France | Retrospective study | Severe, 24 | Moderate, 10 | Virologically confirmed |
| Kalpakci, Y. 2020 | Turkey | Prospective study | Severe, 20 | Non-severe, 20 | Virologically confirmed |
| Kwiecien, I. 2020 | Poland | Retrospective study | COVID-19 X-ray positive, 14 | COVID-19 X-ray negative, 9 | Virologically confirmed |
| Kalicinska, E. 2020 | Poland | Multicentre prospective study | Haematological patients, 16 | Non-haematological patients, 11 | Virologically confirmed |
| Cantenys-Molina, S. 2020 | Spain | Prospective study | Dead, 112 | Alive, 590 | Virologically confirmed |
| Calvet, J. 2020 | Spain | Prospective study | Critical, 13 | Non-critical, 17 | Virologically confirmed |
| Varchetta, S. 2020 | Italy | Retrospective study | Deceased, 15 | Survived, 17 | Virologically confirmed |
| Cui, N. 2020 | China | Retrospective study | Deceased, 13 | Survivors, 118 | Virologically confirmed |
| Yang, P. H. 2020 | China | Prospective study | Patients with pneumonia, 48 | Patients without pneumonia, 22 | Virologically confirmed |
| Fu, Y. Q. 2020 | China | Retrospective study | Death, 14 | Survival, 71 | Virologically confirmed |

**Supplementary table 2. Lymphocyte count of patients with SARS-CoV, MERS-CoV and Influenza. Cell count was shown as mean ± SD and/or median (IQR).**

| Study | Pathogen | N  (Non-severe: severe) | CD3+ T cell count (×10^6^/µL) | | CD4+ T cell count (×10^6^/µL) | | CD8+ T cell count (×10^6^/µL) | | CD4: CD8 ratio | | Reference number (DOI) |
| --- | --- | --- | --- | --- | --- | --- | --- | --- | --- | --- | --- |
|  |  |  | Non-Severe | Severe | Non-Severe | Severe | Non-Severe | Severe | Non-Severe | Severe |  |
| He, Z. 2020 | SARS-CoV | 91: 35 | 737 ± 373 | 591 ± 350 | 418 ± 261 | 303 ± 167 | 299 ± 138 | 269 ± 188 | 1.42 ± 0.59 | 1.42 ± 1.00 | 10.1016/j.ijid.2004.07.014 |
| Wang, N. 2020 | Influenza | 153: 55 | 1169.5 ± 515.7 | 605.5 ± 414.7 | 572.8 ± 259.2 | 317.9 ± 232.7 | 450.0 ± 241.0 | 239.2 ± 160.4 | 1.34 ± 0.53 | 1.38 ± 0.56 | 10.1309/LMRVLJY3BRXLZGE3 |
